# *Lysobacter enzymogenes* prevents *Phytophthora* infection by inhibiting pathogen growth and eliciting plant immune responses

**DOI:** 10.3389/fpls.2023.1116147

**Published:** 2023-01-19

**Authors:** Long Lin, Zixiang Yang, Min Tao, Danyu Shen, Chuanbin Cui, Pingping Wang, Limin Wang, Maofeng Jing, Guoliang Qian, Xiaolong Shao

**Affiliations:** ^1^ College of Plant Protection (State Key Laboratory of Biological interactions and Crop Health; Key Laboratory of Integrated Management of Crop Diseases and Pests), Nanjing Agricultural University, Nanjing, China; ^2^ Department of Plant Pathology, Shaanxi Provincial Tobacco Corporation of CNTC, Xi’an, China

**Keywords:** *Lysobacter*, *Phytophthora*, plant immunity, HSAF, biocontrol

## Abstract

The *Phytophthora* pathogen causes enormous damage to important agricultural plants. This group of filamentous pathogens is phylogenetically distant from fungi, making them difficult to control by most chemical fungicides. *Lysobacter enzymogenes* OH11 (OH11) is a biocontrol bacterium that secretes HSAF (Heat-Stable Antifungal Factor) as a broad-spectrum antifungal weapon. Here, we showed that OH11 could also control a variety of plant *Phytophthora* diseases caused by three major oomycetes (*P. sojae*, *P. capsici* and *P. infestans*). We provided abundant evidence to prove that OH11 protected host plants from *Phytophthora* pathogen infection by inhibiting mycelial growth, digesting cysts, suppressing cyst germination, and eliciting plant immune responses. Interestingly, the former two processes required the presence of HSAF, while the latter two did not. This suggested that *L. enzymogenes* could prevent *Phytophthora* infection *via* multiple previously unknown mechanisms. Therefore, this study showed that *L. enzymogenes* could serve as a promising alternative resource for promoting plant resistance to multiple *Phytophthora* pathogens.

## Introduction

*Phytophthora* belonging to the phylum Oomycota includes a large number of plant pathogens that cause devastating diseases of important agricultural crops (Kamoun et al., 2015). For examples, Potato late blight caused by *Phytophthora infestans* led to the Irish famine in the 1840s and still threatens potato production worldwide to date (Fry, 2008). Soybean root rot caused by *Phytophthora sojae* is one of the most destructive diseases in soybean production (Tyler, 2007). *Phytophthora capsici* is an extremely destructive pathogen with a broad host range that attacks hosts in the Solanaceous, Fabaceae, and most Cucurbitaceae (Lamour et al., 2012). *Phytophthora* shares similar morphology and habitat with fungi, but it is evolutionarily distant from fungi, belonging to the kingdom Stramenopiles (Baldauf et al., 2000; Kamoun et al., 2015). The differences between *Phytophthora* and fungi in terms of genome structure, metabolic pattern, and pathogenic mechanism make *Phytophthora* difficult to control by most fungicides (Latijnhouwers et al., 2003). In this context, environment-friendly biocontrol bacteria are emerging as an important microbial resource for effective control of *Phytophthora* diseases (Sang et al., 2011; de Andrade Lourenco et al., 2022). Members of plant-beneficial genera *Bacillus* and *Pseudomonas* are representatives of such biocontrol bacteria (Caulier et al., 2018). For example, *Bacillus amyloliquefaciens* and *Bacillus subtilis* have been reported to inhibit mycelial growth, cyst germination, and zoospore motility of *P. sojae* (Liu et al., 2019). *Bacillus velezensis* FZB42 antagonizes the growth and virulence of *P*. *sojae* by producing bacilysin (Han et al., 2021). *Pseudomonas aurantiaca* ST-TJ4 inhibits the growth of *Phytophthora cinnamomi* through production of antimicrobial phenazine compounds and volatile organic compounds (VOCs) (Zhang et al., 2022).

The genus *Lysobacter* comprises numerous environmentally ubiquitous biocontrol bacteria with unique ability to prey on other microorganisms by secreting abundant antibiotics, antimicrobial compounds, and lytic enzymes (Puopolo et al., 2018). These powerful weapons enable certain members of *Lysobacter* to efficiently kill the *Phytophthora* pathogens in the laboratory or in the field (Puopolo et al., 2018; Lin et al., 2021). For instance, *Lysobacter antibioticus* HS124 produces 4-hydroxyphenylacetic acid and several lytic enzymes to against *P*. *capsici* mycelial growth (Ko et al., 2009). *Lysobacter capsici* AZ78 generates cyclo (L-Pro-L-Tyr), a 2,5-diketopiperazine that inhibits sporangia development and virulence in *P. infestans* (Puopolo et al., 2014). VOCs from *L. capsici* have been reported to suppress mycelial growth of various fungi and oomycete (Vlassi et al., 2020a; Vlassi et al., 2020b).

*Lysobacter enzymogenes* is the most studied species in the genus *Lysobacter* (Puopolo et al., 2018; Lin et al., 2021). As the name suggested, *L. enzymogenes* can secrete abundant extracellular lytic enzymes (i.e protease and β-1,3-glucanase) that degrade the cell walls of filamentous fungi (Qian et al., 2013). Notably, this bacterium is primarily considered an antifungal agent, because it produces a well-characterized antibiotic metabolite called Heat-Stable Antifungal Factor (HSAF), a novel macrocyclic-lactam compound (Yu et al., 2007). HSAF in *L. enzymogenes* is synthesized by a gene cluster called the HSAF biosynthetic gene operon, in which *lafB* encodes a hybrid polyketide synthase and a nonribosomal peptide synthetase (Li et al., 2014; Wang et al., 2017). HSAF targets filamentous fungi with a unique action mode that disrupts the biosynthesis of fungal cell membrane-associated sphingolipids (Li et al., 2006). Due to its broad-spectrum antifungal activity, HSAF is found to disrupt mycelial growth and spore germination of various fungi such as *Aspergillus nidulans* and *Alternaria alternata* (Li et al., 2006; He et al., 2018). In the field, HSAF shows significant biocontrol activity in controlling wheat Fusarium head blight caused by *Fusarium graminearum* (Zhao et al., 2019). Although we recently reported that seed coatings assembled with HSAF inhibited mycelial growth of the oomycete pathogen *Pythium graminerum* (Ren et al., 2020), biocontrol potential of *L. enzymogenes* and HSAF in controlling *Phytophthora* diseases have not been systematically investigated.

Here, we used *L. enzymogenes* OH11 (herein refers to OH11) as a working model to verify the above viewpoint. We found that OH11 effectively controlled *P. sojae*, *P. capsici* and *P. infestans* on host plants *via* multiple HSAF-dependent and HSAF-independent mechanisms, suggesting this strain may be considered as a promising alternative biocontrol agent against *Phytophthora* diseases in important agricultural crops.

## Results

### *L. enzymogenes* OH11 protected plants against *Phytophthora* infection

**Figure 1:**
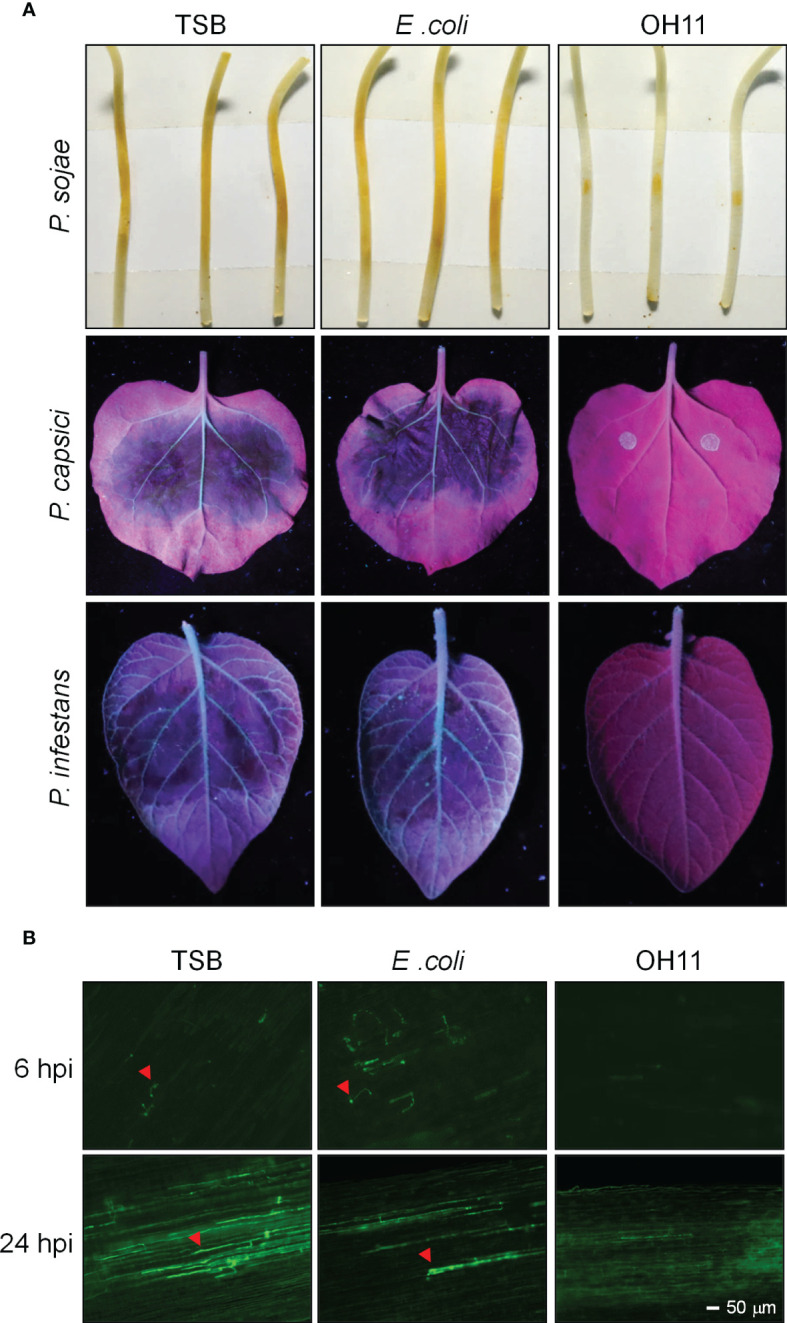
*L. enzymogenes* OH11 inhibited the virulence of *Phytophthora*. **(A)** OH11 inhibited the virulence of *P. sojae*, *P. capsici* and *P. infestans.* A total of 200 *Phytophthora* zoospores were mixed with an equal volume of TSB media, bacterial cultures of *E coli* and OH11, individually. The mixtures were inoculated onto host plants (soybean, *N. benthamiana*, or potato). TSB- and *E. coli*-treated *Phytophthora* had obvious lesions on host plants, while OH11- treated *Phytophthora* revealed no lesion. **(B)** OH11 inhibited infection of GFP-labeled *P. sojae* zoospores. At 6 hpi, TSB- and *E coli*-treated *P. sojae* showed germinated cysts that could efficiently infect soybean cells, whereas OH11-treated *P. sojae* was not able to infect plants. At 24 hpi, TSB- or *E coli*-treated *P. sojae* infected soybean cells, whereas OH11-treated GFP-labeled *P. sojae* showed no invasive hyphae in soybean cells. Red arrows indicate the invasive hyphae of *P. sojae*.

To study the biocontrol ability of *L. enzymogenes* OH11 (OH11) against *Phytophthora* infection, we cultivated OH11 in LB media overnight and transferred the culture to 1/10 TSB (herein refers to as TSB) media for 24 hours to induce HSAF production, as previously described (Qian et al., 2013). The cultures were normalized to OD_600 =_ 1.0 with fresh TSB for further use. *Phytophthora* zoospores were prepared and normalized to 100 zoospores/μL. Afterwards, 200 zoospores of *P. sojae* strain P6497 were mixed with the prepared OH11 culture and inoculated on soybean (*Glycine max*) etiolated seedlings, where both TSB and an irrelevant *E. coli* strain Top10 were used as negative controls. Two days after inoculation, *P. sojae* treated by TSB or *E. coli* caused obvious lesions on soybean seedlings, while OH11 significantly inhibited the virulence of *P. sojae* (**Figure 1A**). We analysed infected soybean seedlings in more detail at the microscopic level with GFP-labelled *P. sojae*. At 6 hpi (hours post inoculation), both TSB- and *E. coli*-treated zoospores had germinated and started to infect soybean, whereas no cyst or hyphae was observed in OH11-treated zoospores. After 24 hpi, the TSB- or *E. coli*-treated *P. sojae* invaded into soybean and grew in soybean cells, while OH11-treated *P. sojae* showed no invasive hyphae (**Figure 1B**), suggesting that OH11 completely inhibited the infection ability of *P. sojae* to soybean cells. Using a similar method, we found that OH11 also inhibited the virulence of *P. capsici* (LT263) on *N. benthamiana* leaves and *P. infestans* (88069) on potato (*Solanum tuberosum*) leaves (**Figure 1A**). These results collectively indicated that *L. enzymogenes* OH11 could effectively prevent infection of its host plants by various *Phytophthora* pathogens.

### *L. enzymogenes* OH11 inhibited mycelial growth of *Phytophthora*

**Figure 2:**
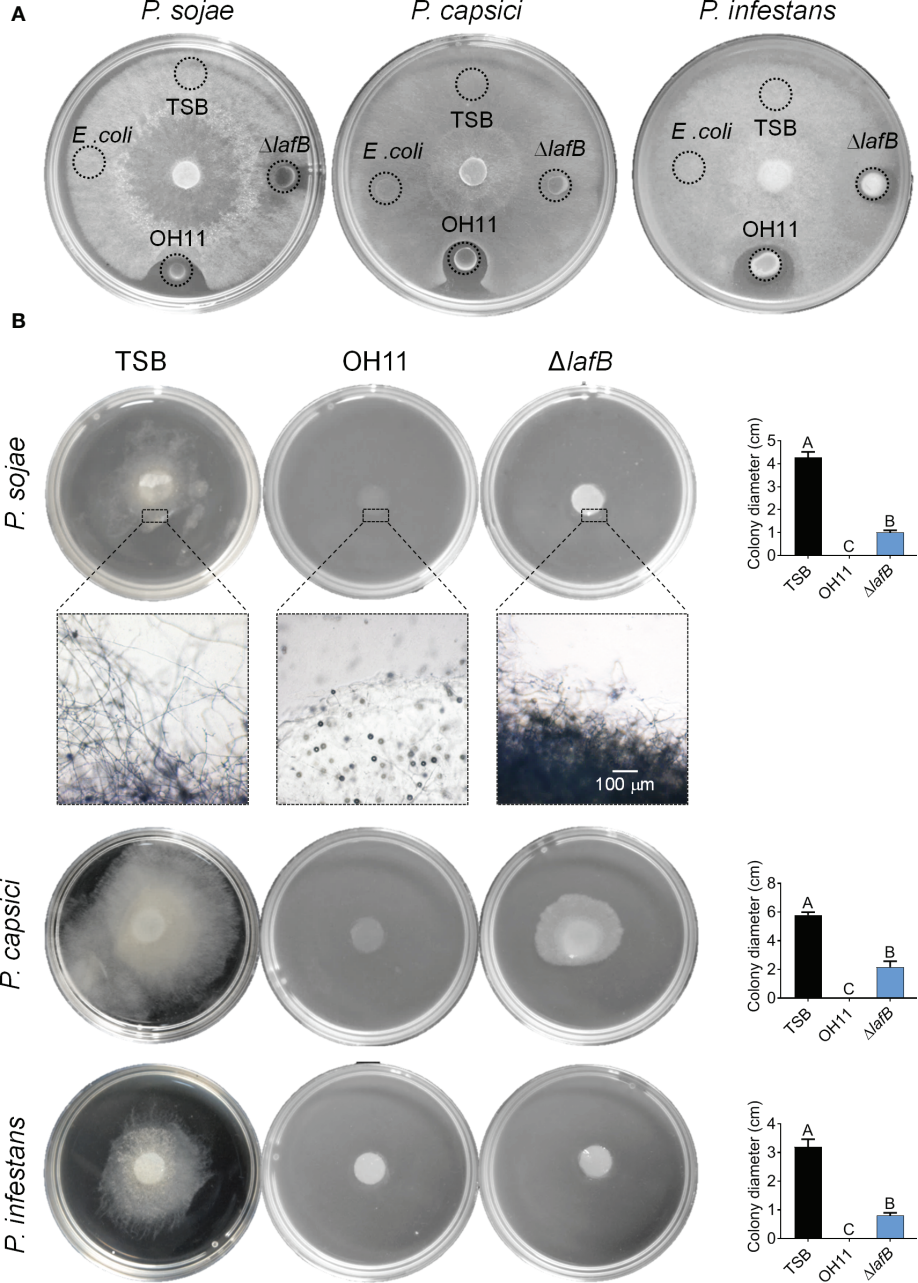
*L. enzymogenes* OH11 inhibited mycelial growth of the *Phytophthora* pathogens. **(A)** Compared with wild-type OH11, the HSAF-deficient mutant Δ*lafB* had less antagonistic activity against *Phytophthora* on solid media. *lafB* is a key gene for HSAF biosynthesis. **(B)** Wild-type OH11 degraded mycelia of *Phytophthora. Phytophthora* hyphal plugs were cultured in liquid media mixed with TSB, OH11, and Δ*lafB*, respectively. OH11 completely inhibited *Phytophthora* mycelial growth and degraded the mycelia in hyphal plugs, while Δ*lafB* inhibited mycelial growth of *Phytophthora*. Three replicates of each sample were analyzed using a one-way ANOVA test.

To understand how OH11 targets *Phytophthora* pathogens to suppress their infection, we first tested whether the antifungal metabolite HSAF is a potentially key factor (Yu et al., 2007). For this purpose, we carried out a plate-based anti- *Phytophthora* assay in which 5 μL cultures of wild-type OH11 or the HSAF-deficient mutant Δ*lafB* generated in a previous study (Wang et al., 2017) were spotted on the surface edge of V8 or RSA agar plates followed by inoculation of three *Phytophthora* pathogens (*P. sojae* P6497, *P. capsici* LT263 and *P. infestans* 88069) in the center of the plate. After incubation for 3 days, we found that wild-type OH11 produced a distinct zone of inhibition around the tested *Phytophthora* colonies, whereas HSAF-deficient mutants almost lost this antagonistic activity (**Figure 2A**).

To confirm the above findings, we carried out similar assays in liquid media in which hyphal plugs of *P. sojae* P6497, *P. capsici* LT263 or *P. infestans* 88069 were mixed with empty V8 broth (negative control) or *L. enzymogenes* cultures, respectively. After 3-day co-inoculation, we found that wild-type OH11 significantly inhibited mycelial growth of the three *Phytophthora* pathogens mentioned above compared with the negative control (**Figure 2B**). Under similar testing conditions, we were surprised to find that the HSAF-deficient mutant Δ*lafB* also showed significant inhibition of mycelial growth compared to the negative control (**Figure 2B**). *Via* microscopic observation represented by *P. sojae*, we further observed that wild-type OH11 could completely degrade mycelia, while HSAF-deficient mutants and negative control (TSB) could not, although Δ*lafB* also effectively inhibited hyphal growth in liquid medium (**Figure 2B**). These results suggested that, while HSAF is important during the antagonistic interaction between *L. enzymogenes* and *Phytophthora* pathogens, other HSAF-independent factors were also involved in this process.

### *L. enzymogenes* OH11 inhibited *Phytophthora* cyst germination

**Figure 3:**
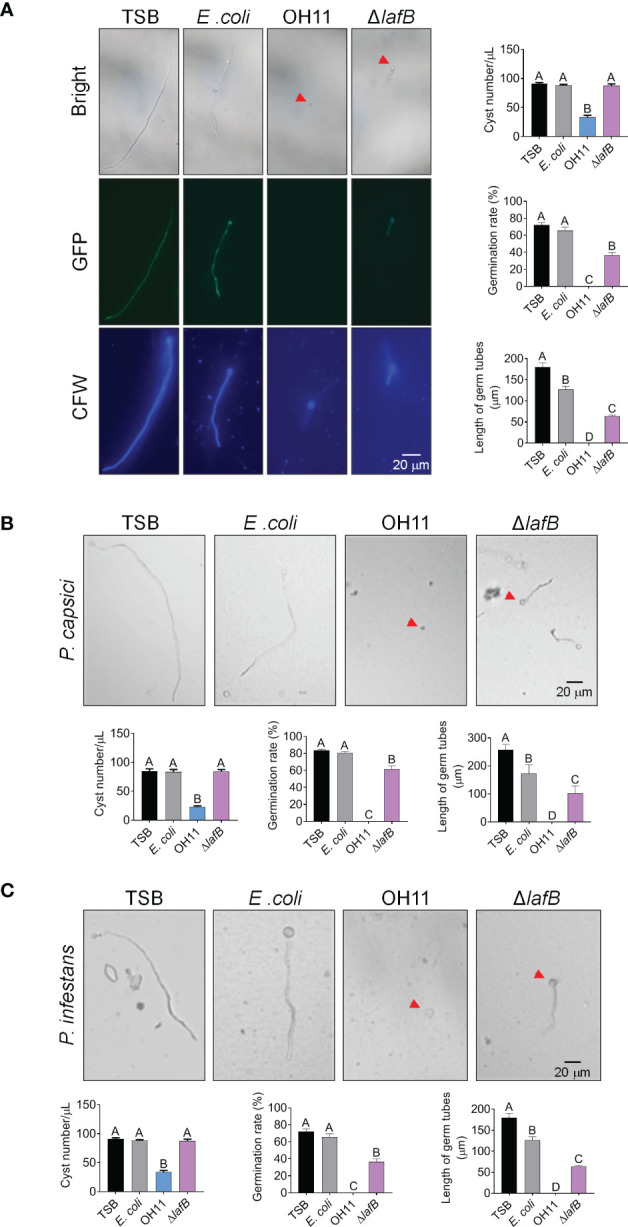
*L. enzymogenes* OH11 inhibited cyst germination of *Phytophthora*. **(A)** Wild-type OH11 degraded *P. sojae* cysts, whereas the HSAF-deficient mutant Δ*lafB* did not, but inhibited cyst germination. GFP-labeled *P. sojae* cysts were mixed with TSB, *E coli*, OH11, or Δ*lafB*, and cultured at 25°C for 6 hours. Compared to TSB control, OH11 treatment completely inhibited cyst germination in *P. sojae* and the number of cysts after OH11 treatment was significantly reduced. At the same time, both the germination rate and the germinated tube length of *P. sojae* were significantly reduced after Δ*lafB* treatment. Three replicates of each sample were analyzed using a one-way ANOVA test. Red arrows indicate *P. sojae* cysts. WT-treated cysts showed a lower GFP signal, indicating that WT-treatment resulted in cell death of *P. sojae* cysts. Cysts were stained with chitin-binding CFW to make them clearer to observe. **(B, C)** WT degraded *P. capsici* **(B)** and *P. infestans* **(C)** cysts, whereas Δ*lafB* only inhibited germination of the corresponding cysts. *P. capsici* and *P. infestans* cysts were mixed with TSB, *E coli*, OH11, and Δ*lafB*, respectively, and cultured at 25°C (*P. capsici*) or 18°C (*P. infestans*) for 6 hours (*P. capsici*) or 12 hours (*P. infestans*). Three replicates of each sample were analyzed with a one-way ANOVA test. Red arrows indicate *P. capsici* or *P. infestans* cysts.

Besides mycelial growth, cyst germination is another key step preceding *Phytophthora* infection (Judelson and Blanco, 2005). We therefore investigated whether *L. enzymogenes* OH11 could inhibit cyst germination of *Phytophthora* pathogens in an HSAF-dependent and/or HSAF-independent manner. We carried out a corresponding assay to address this issue using GFP-labeled *P. sojae* as a representative. We mixed *P. sojae* cysts with cultures of wild-type OH11 or HSAF-deficient mutant Δ*lafB* in liquid broth, followed by microscopic observation. As shown in **Figure 3A**, approximately 80% of *P. sojae* cysts were degraded by OH11 after 6 hours of co-incubation compared to the negative control (TSB), and almost all remaining cysts showed undetectable GFP signals, indicating that *L. enzymogenes* was able to kill *P. sojae* cysts instantly. This killing effect appeared to be specific, as another negative control (*E. coli*) failed to do so. Under similar testing conditions, although Δ*lafB* failed to directly kill *P. sojae* cysts, this mutant also significantly reduced germination rate and germ tube length of *P. sojae* (**Figure 3A**). After 24 hours of co-incubation, wild-type OH11 completely digested cysts, whereas Δ*lafB* only inhibited cyst germination (**Supplemental Figure S1**). Similar findings were also observed when *P*. *capsici* and *P. infestans* cysts were applied (**Figures 3B, C**). Together, these results indicated that *L. enzymogenes* OH11 had the ability to directly degrade *Phytophthora* cysts, and that HSAF was involved in this process. In the absence of HSAF, *L. enzymogenes* might also inhibit cyst germination through other uncharacterized factors/pathways.

### Transcriptomic profiling revealed regulation of *P. sojae* cyst genes following stimulation with *L. enzymogenes* OH11

**Figure 4:**
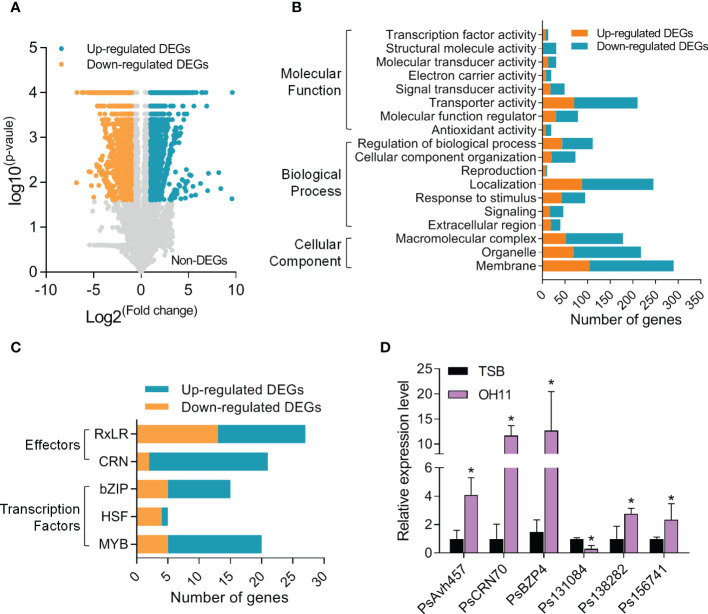
*L. enzymogenes* OH11 treatment resulted in global changes in *P. sojae* cyst gene expression. **(A)** Volcano plot of OH11-treated *P. sojae* DEGs (differentially expressed genes) compared to medium (TSB) control. Orange spots indicate the 1741 up-regulated genes; blue spots indicate the 2176 down-regulated genes; grey spots list those non-differentially expressed genes. **(B)** Number of DEGs categorized into different GO terms. **(C)** Number of DEGs categorized into different effector and transcription factor families. **(D)** Expression levels of representative DEGs verified by qRT-PCR. Three replicates of each sample were analyzed with a t-test. Asterisks indicate significant differences (P < 0.01).

The interesting findings above prompted us to explore the underlying mechanisms at the molecular level. To this end, we carried out an RNA-seq assay to identify *P. sojae* cyst genes whose transcription could be significantly altered by *L. enzymogenes* OH11 treatment. To facilitate this assay and considering that wild-type OH11 can degrade the cysts in liquid medium, we therefore depleted OH11 cells by filter and treated *P. sojae* cysts with the cell-free supernatant that exhibited resistance to *P. sojae* activity similar to total OH11 culture (**Supplemental Figure S2**). The results of RNA-seq assay showed that among the 13014 expressed *P. sojae* genes, 1741 genes were up-regulated and 2176 genes were down-regulated in the OH11-treated *P. sojae* compared with the negative control (TSB-treated) (**Figure 4A**). Details of differentially expressed genes (DEGs) were listed in **Supplemental Table S1**. The functions of DEGs were enriched in basal metabolism, including translation initiation, RNA binding, and amino acid metabolism (**Supplemental Figure S3**). Meanwhile, a large number of genes involved in signal transduction (46 in total number), stimulus response (95 in total number), antioxidation (20 in total number), and transporting (210 in total number) were also differentially expressed (**Figure 4B**). Considering that *Phytophthora* can secrete effectors to subvert plant immunity and promote pathogen infection (Wang and Wang, 2018), and several transcription factors (TFs) of bZIP, MYB and HSF families are key transcriptional regulators of during *Phytophthora* cyst germination (Zhang et al., 2012; Ye et al., 2013; Sheng et al., 2015), we also investigated whether these genes were included in the RNA-seq data. As shown in **Figure 4C**, we found that the expression levels of 27 RxLR effector genes and 21 CRN effector genes as well as 15 bZIP TFs, 5 HSF TFs and 20 MYB TF genes were significantly changed when *P. sojae* interacted with *L. enzymogenes* OH11. In addition, the expression levels of *PsAvh457* (RxLR effector), *PsCRN70* (CRN effector), *PsBZP4* (bZIP TF), *Ps138282* (MYB TF), *Ps156741* (homolog of apoptosis-inducing centromere protein) and *Ps131084* (cell wall endo-beta-1, 3-glucanase) were verified by quantitative real-time PCR (qRT-PCR) (**Figure 4D**). These results provided molecular evidence supporting the conclusion that *L. enzymogenes* prevented *P. sojae* infection by attacking the cysts.

### *L. enzymogenes* OH11 induced immune response in plants

**Figure 5:**
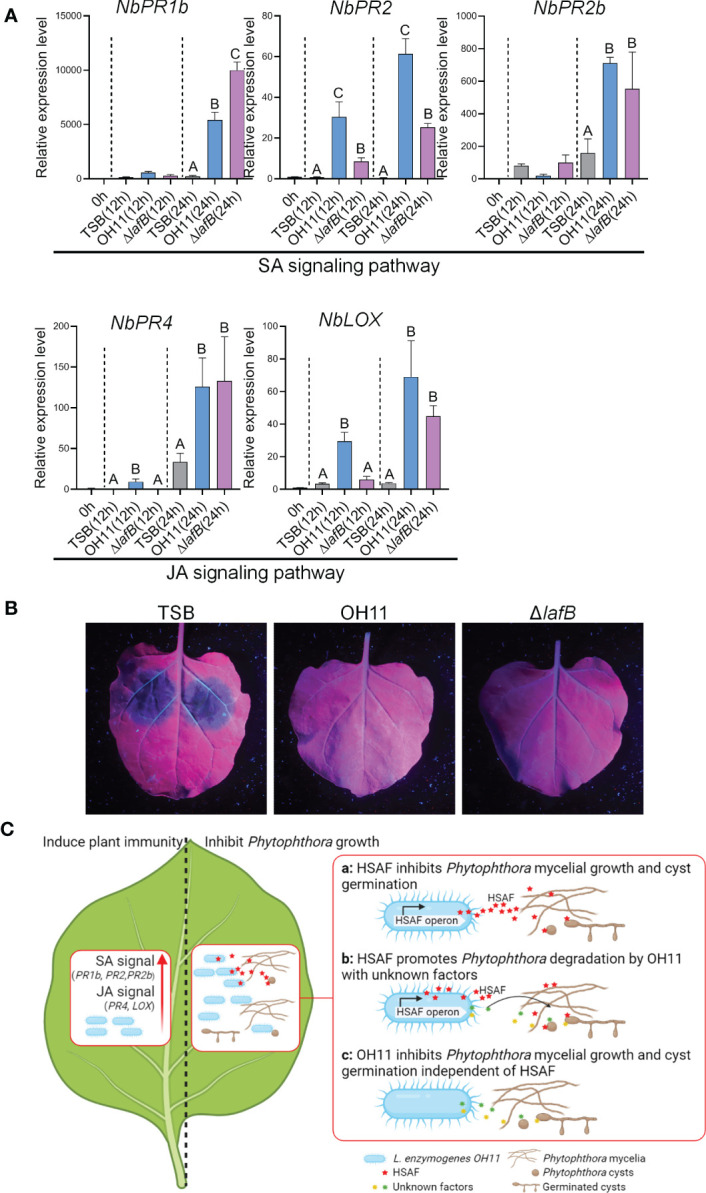
*L. enzymogenes* OH11 treatment elicited immunity responses in *N. benthamiana*. **(A)** Treatment of wild-type OH11 and HSAF-deficient mutant Δ*lafB* up-regulated expression levels of defense-related genes in *N. benthamiana* leaves. Gene expression levels were measured 12 and 24 hours after treatment, respectively. The relative expression level of untreated leaves (0h) was set to 1.0. Three replicates of each sample were analyzed using a one-way ANOVA test. **(B)** Pretreatment with WT and Δ*lafB* prevented infection of *N. benthamiana* leaves from *P. capsici*. *P. capsici* zoospores were inoculated onto *N. benthamiana* leaves 24 hours after pretreatment. TSB-treated *N. benthamiana* leaves showed obvious lesions, while OH11- and Δ*lafB-*treated leaves did not show any lesion. **(C)** A working model showing how *L. enzymogenes* prevented *Phytophthora* infection. When *L. enzymogenes* colonized plant surfaces through biofilms, it induced an immune response (left panel) to protect plants from pathogen infection by activating genes involved in SA and JA signaling. On the right, *L. enzymogenes* could directly target and inhibit the growth of *Phytophthora*. HSAF is a key weapon of OH11 that inhibited *Phytophthora* mycelial growth and cyst germination **(a)**. HSAF also enhanced the degradation activity of OH11 on *Phytophthora* mycelia and cysts by unknown factors **(b)**. When HSAF was blocked by environmental factors, OH11 could also inhibit *Phytophthora* mycelial growth and cyst germination through other unknown factors **(c)**. The symbol ABCs indicate significant differences (P < 0.01).

In addition to directly targeting pathogens, eliciting host plant immune response is another common biocontrol strategy shared by plant-beneficial *Pseudomonas* and *Bacillus* species (Yu et al., 2022). We were therefore also interested in finding out whether *L. enzymogenes* could use this similar strategy to prevent *Phytophthora* infection. To address this question, we selected *P. capsici*-*N. benthamiana* interaction system as a working model. We first treated leaves of *N. benthamiana* with TWEEN-20 to promote bacterial adhesion to plant surfaces (Paul et al., 2012), and then sprayed cultures of wild-type OH11 and Δ*lafB* on the leaves. Meanwhile, empty TSB broth was used as a negative control. After 12 hours and 24 hours of treatment, we examined the expression levels of those well-characterized plant defence-related genes in *N. benthamiana* that serve as molecular indicators of plant immune responses, as previously described (Yang et al., 2021). The result showed that both wild-type OH11 and Δ*lafB* induced the expression of *NbPR1b* (Pathogenesis-related protein 1b), *NbPR2*, *NbPR2b*, *NbPR4* and *NbLOX* (Lipoxygenase) at 24h. At 12 h, OH11 induced higher levels of *NbPR2*, *NbPR4* and *NbLOX* gene expression compared with Δ*lafB* (**Figure 5A**). Among them, *NbPR1b*, *NbPR2* and *NbPR2b* are known maker genes of the salicylic acid (SA) signaling pathway, while *NbPR4* and *NbLOX* are well characterized to participate in the jasmonic acid (JA) signaling pathway (Zhang et al., 2015; Yu et al., 2022). These results indicated that OH11 could elicit plant immune response in a HSAF-independent manner by activating the expression of defense genes involved in the SA and JA signaling pathways. To validate this point, we conducted another plant-based biocontrol assay. We inoculated *P. capsici* zoospores on *N. benthamiana* leaves at 24 hours after *L. enzymogenes* treatment and found that both wild-type OH11 and Δ*lafB* effectively inhibited the infection of *N. benthamiana* by *P. capsici* (**Figure 5B**).

## Discussion

Oomycetes represented by *Phytophthora* are phylogenetically distant from fungi, and are difficult to control by most antifungal agents (Latijnhouwers et al., 2003). On the other hand, most *Phytophthora* species have sizeable, repeat-rich genomes. The variability of the genome leads to fast adaptive evolution, which is conducive to the rapid evasion of host resistance genes of *Phytophthora* and the development of resistance to chemical fungicides (Leesutthiphonchai et al., 2018). Biological control is a sustainable and environmentally friendly solution for managing *Phytophthora* diseases. Most biocontrol activity against the bacterial *Phytophthora* is measured by antagonistic activity against the mycelium. Previously, the studies on the interaction between *Lysobacter* and *Phytophthora* were mainly limited to the inhibition of mycelial growth, while zoospores are the main carrier of *Phytophthora* dispersal and infection (Judelson and Blanco, 2005). In the field, zoospores can sense chemical signals released by host plant and swim chemotactically to the host. After attaching to the host, zoospores will lose their flagella and form walled cyst. The cyst will then germinate and extend a germ tube to start infection (Judelson and Blanco, 2005). In addition to inhibiting mycelium growth, this study also reported that *L. enzymogenes* OH11 could degrade cysts and inhibited infection by various *Phytophthora*, making OH11 a promising agent for field control of *Phytophthora* pathogens.

HSAF is a polycyclic tetramate macrolactam synthesized and secreted by *L. enzymogenes* with broad-spectrum inhibitory activity against filamentous fungi (Lou et al., 2011). Compared with wild-type OH11, the HSAF-deficient mutant Δ*lafB* showed defective activity against *Phytophthora*, consistent with the earlier finding that HSAF has a direct inhibitory effect on *Phytophthora* mycelium growth (Li et al., 2008). This was also consistent with earlier reports that *L. enzymogenes* C3 mutants unable to release HSAF shows reduced activity in inhibiting fungal mycelial growth and spore germination (Yu et al., 2007; Li et al., 2008). Interestingly, unlike wild-type OH11, Δ*lafB* failed to degrade mycelia and cysts of *Phytophthora*, indicating that HSAF had an additional previously unknown role beyond its direct anti-*Phytophthora* effect.

HSAF targets fungal sphingolipids biosynthesis by blocking ceramide synthase in *A. nidulans*, resulting in arrest of hyphal tip elongation (Li et al., 2006). HSAF also induces apoptosis in *Candida albicans* by triggering the generation of reactive oxygen species and binding to β-tubulin (Ding et al., 2016). Besides, HSAF treatment disrupts multiple signaling networks and fundamental cellular metabolisms in *A. alternata* (He et al., 2018). Our RNA-seq data showed that OH11 treatment resulted in differential expression of 20 antioxidant-related genes and one apoptotic-inducing centromere protein homolog in *Phytophthora*. These *Phytophthora* gene expression changes might be due to HSAF-induced apoptosis and ROS (reactive oxygen species) burst, providing clues for future investigations on the mode of action of HSAF against the *Phytophthora* pathogens.

It is interesting to observe the direct digestion of *Phytophthora* mycelia and cysts by *L. enzymongenes* OH11. Although we do not have a definitive answer to explain how this occurs, abundant cell wall-targeting cleavage enzymes (i.e. protease and β-1,3-glucanase) secreted by *L. enzymogenes* might be involved in this process. In agreement, previous reports have shown that HSAF-deficient mutants produce some unidentified thermostable antifungal factors that degrade fungal mycelia (Odhiambo et al., 2017). Therefore, these unidentified factors might also have activity against *Phytophthora*.

Interactions between biocontrol bacteria and phytopathogenic fungi/oomycetes are complex. Bacteria attack fungi/oomycetes with various weapons, and fungi/oomycetes defend bacteria attack through different patterns (Tomada et al., 2017; Wang et al., 2020). 15 bZIP TFs, 5 HSF TFs and 20 MYB TFs of *P. sojae* showed differential expression levels during interacting with *L. enzymogenes*. Disorder of gene expression regulation may involve in degradation of *Phytophythora* cysts. On the other hand, differentially expression of TFs may be a feedback response to defend the OH11 inhibition. *Phytophthora* pathogens cohabited with other microbes and showed anti-bacteria activity to gain ecological adaptation in the field. Effectors secreted by *Phytophthora* not only subvert plant immunity and promote pathogen infection, but also interfere bacteria physiology to compete with bacteria in the same habitat and defend attack from biocontrol bacteria (Wang and Wang, 2018; Wang et al., 2020). It is noteworthy that expression of 14 RxLR effectors and 19 CRN effectors were induced when *P. sojae* interacting with *L. enzymogenes*. These effectors may involve in inhibiting or defending the OH11 inhibition.

Many biocontrol bacteria, represented by *Bacillus* and *Pseudomonas*, can stimulate plant immune responses and help plants acquire broad-spectrum resistance to pathogens (Yu et al., 2022). Cellular components of these bacteria, such as flagellin and lipopolysaccharides (LPS), can be recognized by plant cell surface receptors to generate pattern-triggered immunity (PTI) (Zamioudis and Pieterse, 2012). In addition to cellular components, some secondary metabolites produced by beneficial bacteria also induce plant defense responses (Yu et al., 2022). For example, pyocyanin produced by *Pseudomonas* activates plant defense responses by inducing the accumulation of H_2_O_2_ (De Vleesschauwer et al., 2006). *L. enzymogenes* C3 were reported to induce common resistance of tall fescue and wheat to fungi pathogens, and this immune induction was independent of the strain’s antifungal ability (Kilic-Ekici and Yuen, 2003). We found that *L. enzymogenes* OH11 could induce the expression levels of defence-related genes involved in SA and JA signalling pathways, which appeared to be independent of HSAF, but seemed to enable *L. enzymogenes* to protect host plants from pathogen infection under natural conditions where HSAF generation was hindered by environmental factors. It is also noteworthy that *L. enzymogenes* is a non-flagellated bacterium that does not produce flagellin (Xia et al., 2018). Instead, it displays twitching motility driven by type IV pili (T4P) on solid surface and is essential for the bacterium to form biofilms that facilitate its colonization on plants (Xia et al., 2018). Using T4P, *L. enzymogenes* can secrete pili that are the first to contact the plant surface. Therefore, it is possible that non-flagellated *L. enzymogenes* used type IV pilin as the initial signal to elicit plant immune response, a hypothesis we are now testing in our laboratory.

In conclusion, we reported that *L. enzymogenes* OH11 could protect plants against *Phytophthora* infection by inducing plant immune responses through activation of SA and JA signaling pathways. OH11 also protected plants by directly targeting *Phytophthora*, which not only secreted HSAF to inhibit *Phytophthora* mycelium growth as previously described (Li et al., 2008), but also directly degraded *Phytophthora* mycelia and cysts in a HSAF-dependent manner. Furthermore, OH11 could also inhibit *Phytophthora* mycelial growth and cyst germination in the absence of HSAF through unknown factors (**Figure 5C**). These multiple mechanisms made *L. enzymogenes* OH11 an ideal bacterium for the control of various *Phytophthora* diseases.

## Materials and methods

### Strains and growth conditions

The strains used in this study are listed in **Supplementary Table S2**. *P. sojae* strain P6497 and *P. capsici* strain LT263 were routinely cultured on 10% V8 media at 25°C, while *P. infestans* strain 88069 was routinely cultured on RSA media at 18°C. Soybean cultivar Hefeng47 was grown at 25°C in the dark for 4 days in environmentally controlled growth chamber, and etiolated soybean seedlings were used for infection assays. *N. benthamiana* was grown at 25°C in a growth chamber with a 16 h light and 8 h dark photoperiod, and 4-week-old leaves were selected for infection assay. Potato cultivar Desiree was grown at 22°C in a growth chamber with a 16 h light and 8 h dark photoperiod, and 4-week-old leaves were selected for infection assay. Bacteria strains were grown overnight in LB media at 28°C and then transferred to 10% TSB media for 24 h at 28°C to induce HSAF production.

For mycelial growth assays on solid media, 5 × 5-mm hyphal plugs of *P. sojae* or *P. capsici* were inoculated onto 10% V8 medium plates at 25°C, while *P. infestans* hyphal plugs were inoculated onto RSA medium plates at 18°C. When the fresh mycelia grew to a suitable size (3 cm in diameter), 5 μL of bacterial culture (diluted to an OD600 = 1.0) was inoculated to the edge of the plates. Antagonistic activity was measured by an inhibition zone around the colony. For mycelial growth assays in liquid media, 5 × 5-mm hyphal plugs of *P. sojae* or *P. capsici* were inoculated onto the plates containing 4 mL 10% V8 liquid medium at 25°C, while *P. infestans* hyphal plugs were inoculated onto plates containing 4 mL PEA liquid medium at 18°C. 1 mL of bacterial culture (diluted to an OD600 = 1.0) was inoculated onto the plates. Antagonistic activity was measured by the size of *Phytophthora* colony. Mycelia colonies were observed with a microscope (Zeiss Axio Observer 3).

### Plant infection assays

To induce zoospore production, mycelia of *P. sojae* or *P. capsici* were cultured in 10% liquid V8 medium for 3 days, washed with sterile water, and incubated at 25°C until zoospores were produced. To induce zoospore production of *P. infestans*, mycelia was grown on RSA medium plate at 18°C for 14 days. The sporangia were scraped into sterile water and incubated at 4°C until zoospores were released. Zoospores were diluted to 100 zoospores/μL and mixed with an equal volume of bacterial cultures (diluted to an OD600 = 1.0). A final mixture containing 200 zoospores were inoculated onto the host plants. Infected soybean and *N. benthamiana* were incubated in the dark at 25°C for 2 days before observation. Infected potatoes were incubated in the dark at 18°C for 3 days before observation. Lesions of plant leaves were observed under UV light. Infection details of GFP-labelled *P. sojae* zoospores were observed with a microscope (Zeiss Axio Observer 3).

To examine the immune response of *N. benthamiana*, leaves of *N. benthamiana* were quickly soaked into 0.5% (v/v) TWEEN-20 and washed with H_2_O to remove TWEEN-20. The TSB, OH11 and Δ*lafB* cultures were then sprayed on the leaves individually. Treated leaves were incubated at 25°C in the dark before sampling. To detect immune gene expression, leaf samples were collected 12 and 24 hours after treatment. For the infection assay, 200 P*. capsici* zoospores were inoculated onto *N. benthamiana* leaves 24 hours after treatment. Infected leaves were incubated at 25°C in the dark for 2 days before observation.

### Cyst germination assays

*Phytophthora* zoospores were induced and harvested into 1.5 mL tubes. Tubes containing 100 zoospores/μL in a 500 μL suspension were vortexed for 90 s to induce cyst formation. 500 μL cyst suspension were mixed with 500 μL 10% V8 medium and 500 μL bacterial culture (diluted to an OD600 = 1.0), and then incubated at 25°C to observe the germination of *P. sojae* or *P. capsici*. 500 μL *P. infestans* cyst suspension were mixed with 500 μL PEA medium and 500 μL bacterial culture (diluted to an OD600 = 1.0), and then incubated at 18°C to observe the germination, which was observed with a microscope (Zeiss Axio Observer 3). GFP-labeled *P. sojae* cysts were stained with 1 μg/mL chitin-binding CFW (Caleofluor White) for 5 min to make cysts clearer to observe.

### Transcriptome sequencing and analysis

For RNA-seq sampling, bacterial culture of *L. enzymogenes* OH11 were collected and diluted to a density of 1.0 at 600 nm. The bacteria culture was then centrifuged and filtered through a 0.22-μm filter to remove OH11 cells. *P. sojae* cysts were diluted to 100 cyst/µL with sterile ddH_2_O. Bacterial suspension and cysts were mixed in a 1:1 ratio and cultured for 6 hours at 25°C prior to sampling. TSB media-treated cysts were used as negative controls. Three replicates of each sample were collected and sent to BGI Genomics Corporation for RNA-seq. *P. sojae* RNA was extracted using the TRIzol^®^ method following the manufacturer’s protocol. RNA-seq was conducted using the DNBseq platform and 100-bp paired-end modules.

Raw reads were filtered for subsequent analysis by removing reads containing adapter, poly-N, and low-quality reads. Clean reads were mapped to the genome of *P. sojae* (v1.1 for isolate P6497) using Tophat with up to two mismatches. Mapped reads were quantified using the Cufflinks program, and transcript levels for each gene were quantified as RPKM (reads per kilobase transcript length per million reads mapped). Differentially expressed genes were identified using featureCounts software, and log2 fold change (log2FC) values and adjusted P-values were calculated using DESeq2 software. Genes with adjusted P-value < 0.05 and absolute log2FC ≥1 were considered differentially expressed.

### Quantitative real-time PCR assay

For qRT-PCR assays, cDNA was synthesized with PrimeScript First Strand cDNA Synthesis Kit (TaKaRa Bio Inc.) following the manufacturer’s protocol. qRT-PCR was performed in a reaction mixture of 20 μL of SYBR Premix ExTaq (TaKaRa Bio Inc.) following the manufacturer’s protocol. PCR was performed on an ABI Prism 7500 Fast Real-Time PCR System (Applied Biosystems Inc.) The *P. sojae* actin gene (*Psactin*, Ps108986) was used as a constitutively expressed endogenous control for *P. sojae*. The *N. benthamiana* elongation factor 1-alpha gene (*NbEF1α*) was used as endogenous control for *N. benthamiana*. Primers used in this study are listed in **Supplementary Table S3**.

## Data availability statement

The RNA-seq data presented in the study are deposited in the NCBI database, accession number PRJNA909446.

## Author contributions

GQ and XS conceived the project and designed experiments. LL, ZY, MT, CC and PW carried out experiments. LL, DS, LW, GQ and XS analyzed data and prepared figures and tables. LL, MJ, GQ and XS wrote and revised the manuscript. All authors contributed to the article and approved the submitted version.

## Supplementary material

Supplementary Figure 1
*L. enzymogenes* OH11 completely digested the cysts of *P. sojae* 24 hours after treatment.

Supplementary Figure 2
*L. enzymogenes* OH11 supernatant inhibited cyst germination of *P. sojae* 6 hours after treatment.

Supplementary Figure 3 GO enrichment of *Phytophthora* cyst DEGs treated with OH11.
