# Supplementary figures and images for: Lysobacter enzymogenes prevents Phytophthora infection by inhibiting pathogen growth and eliciting plant immune responses
Source: Front Plant Sci. 2023 Jan 19;14:1116147. doi: 10.3389/fpls.2023.1116147 (PMC9892905; doi:10.3389/fpls.2023.1116147)

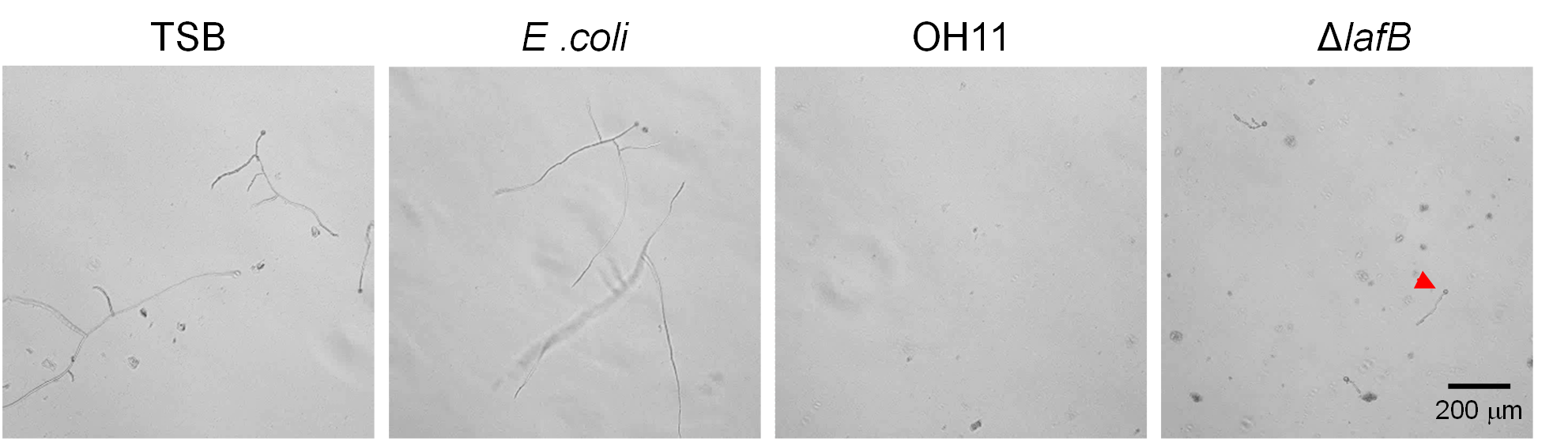

Supplement: Supplementary Figure 1 — L. enzymogenes OH11 completely digested the cysts of P. sojae 24 hours after treatment. [file Image_1.tif]

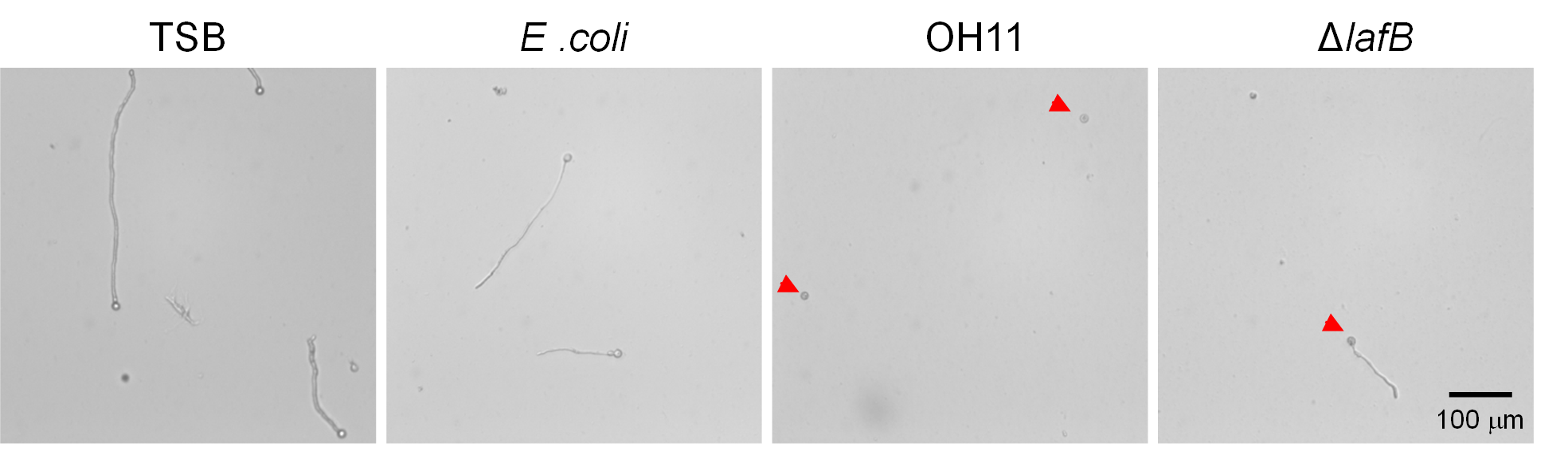

Supplement: Supplementary Figure 2 — L. enzymogenes OH11 supernatant inhibited cyst germination of P. sojae 6 hours after treatment. [file Image_2.tif]

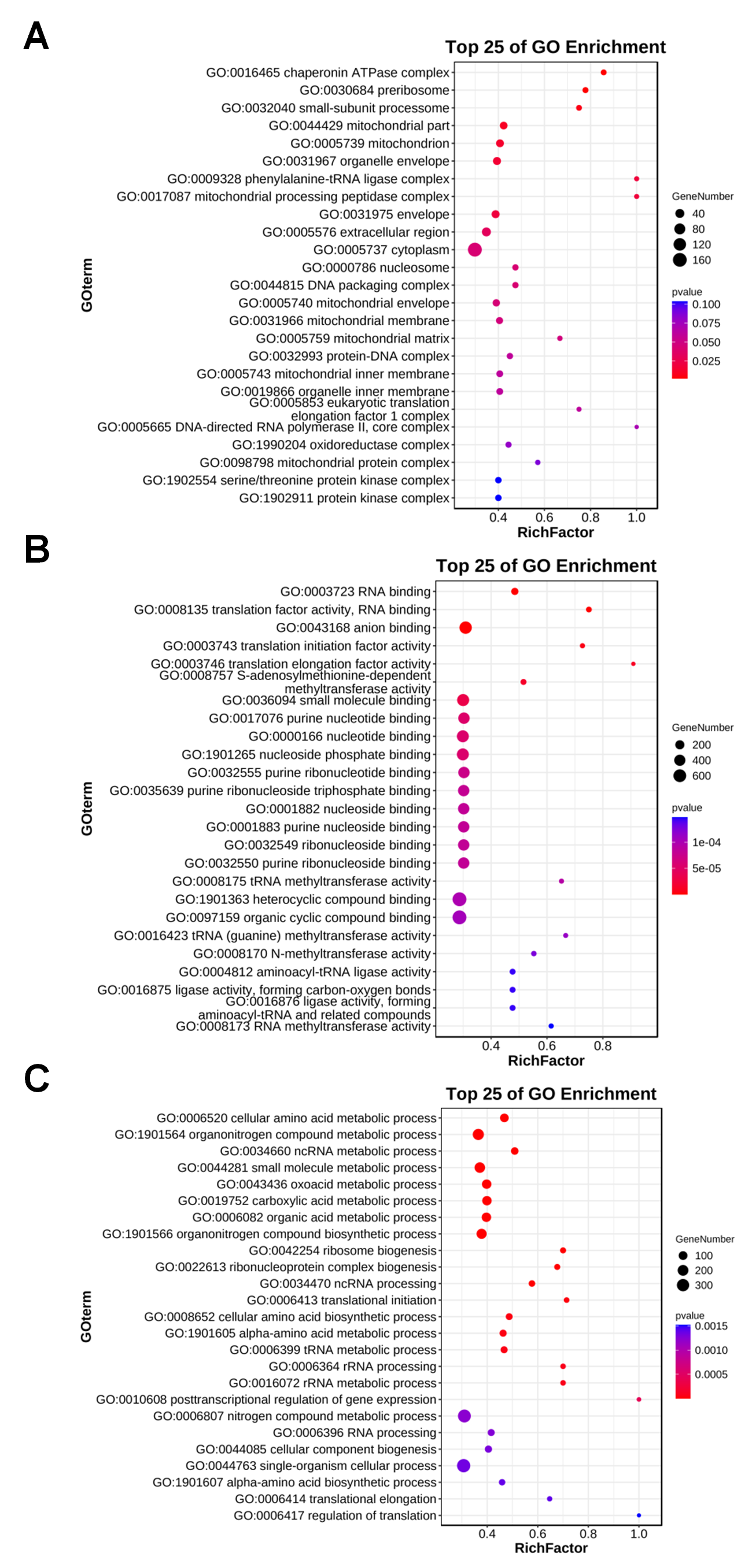

Supplement: Supplementary Figure 3 — GO enrichment of Phytophthora cyst DEGs treated with OH11. [file Image_3.tif]
